# Automatic Labeling of Special Diagnostic Mammography Views from Images and DICOM Headers

**DOI:** 10.1007/s10278-018-0154-z

**Published:** 2018-11-21

**Authors:** Dmytro S. Lituiev, Hari Trivedi, Maryam Panahiazar, Beau Norgeot, Youngho Seo, Benjamin Franc, Roy Harnish, Michael Kawczynski, Dexter Hadley

**Affiliations:** 1 Institute for Computational Health Sciences, University of California, San Francisco, 550 16th Street, San Francisco, CA USA; 2 Department of Radiology and Biomedical Imaging, University of California, San Francisco, 185 Berry St., San Francisco, CA 94143-0946 USA

**Keywords:** Machine learning, Convolutional neural networks, DICOM, Mammography

## Abstract

Applying state-of-the-art machine learning techniques to medical images requires a thorough selection and normalization of input data. One of such steps in digital mammography screening for breast cancer is the labeling and removal of special diagnostic views, in which diagnostic tools or magnification are applied to assist in assessment of suspicious initial findings. As a common task in medical informatics is prediction of disease and its stage, these special diagnostic views, which are only enriched among the cohort of diseased cases, will bias machine learning disease predictions. In order to automate this process, here, we develop a machine learning pipeline that utilizes both DICOM headers and images to predict such views in an automatic manner, allowing for their removal and the generation of unbiased datasets. We achieve AUC of 99.72% in predicting special mammogram views when combining both types of models. Finally, we apply these models to clean up a dataset of about 772,000 images with expected sensitivity of 99.0%. The pipeline presented in this paper can be applied to other datasets to obtain high-quality image sets suitable to train algorithms for disease detection.

**Electronic supplementary material:**

The online version of this article (10.1007/s10278-018-0154-z) contains supplementary material, which is available to authorized users.

## Introduction

Considerable attention in biomedical image research has been devoted towards applying deep learning to digital mammography [1]. However, most research focuses on readily available, manually curated datasets of limited size (on the order of magnitude of hundreds to thousands of images) [2, 3]. In contrast to for example cardiological imaging, where progress has been made in applying deep learning for curation of imaging datasets [4], the task of automating construction of robust mammography datasets from existing medical records remains largely unaddressed in the literature. Developing new datasets would expand the number of relevant clinical questions that could be answered, enable testing of existing models on different patient populations, and increase their accuracy. Given about 40 million mammograms are performed annually in the USA [5], accurate and automatic normalization of these data remains a large-scale problem for deep learning precision mammography interpretation.

We began by extracting 772,367 mammographic images that were obtained at our institution between 2000 and 2016, with the goal of assembling this raw clinical data into a large and unbiased research dataset suitable for training machine learning models for mammography. Our mammography machine learning efforts focus on full-field digital mammograms; thus, we needed a methodology to separate out special diagnostic views, such as stereotactic, spot, magnified, and wire localizations. This is important because special diagnostic views contain a disproportionate number of cancers compared to full-field views which severely biases any deep learning algorithm towards identifying artifacts of specialized views rather than features of any malignancy itself. Therefore, these specialized views must be eliminated from the dataset before model development. Although many mammograms contain explicit DICOM headers identifying the type of view, we learned that these were not reliable as a sole indicator. Therefore, we developed a novel method combining imaging and DICOM header data to reliably classify the views of mammographic images in our dataset. We believe that this is an essential contribution to the field of deep learning precision mammography interpretation, opening the door to automatic cleaning of large scale datasets.

## Materials and Methods

### Dataset

In order to build a clean and non-biased dataset, we use two approaches to identify special diagnostic views: (1) by using DICOM headers and (2) by using images directly. From our database of 772,367 images, we first manually labeled 4000 randomly selected images. Resulting subset contained 3406 full-field mammograms and 594 special views (24 stereotactic, 6 wire localization, 9 specimen views, and the remainder being spot or magnification views). We split the dataset into training, validation, and holdout sets in a ratio of 4:1:1, respectively.

### Header-Based Prediction

For DICOM-based prediction, the headers were extracted using a script based on the “dicom” Python package. Fields with more than 10% missing values and fields indicating date/time, device identifiers or demographic information were omitted. The values were normalized by filling in missing values of numeric fields (such as KVP, BodyPartThickness, DetectorActiveDimension) with their median values and storing a flag variable for missing entries. The categorical variables (such as Grid, ViewCodeValue, Manufacturer) were one-hot encoded (i.e., each categorical variable was transformed into a sub-table with columns corresponding to its possible values). We leveraged three implementations of gradient boosting machines (“gbm” and “xgbTree” from “caret” package [6] and “gbmt” from “gbm3” package), RPART (from “caret”), and Elastic Net logistic regression model (from “glmnet” R package [7]). The header-based model selection was based on grid search of L1/L2 penalty ratio and built-in search for overall penalty strength (λ). The hyperparameters were tuned in fivefold cross-validation within the train set.

### Image-Based Prediction

Additionally, we leveraged a convolutional neural network model based on Inception v3 [8] to predict views from images. In order to accelerate training, we used an abridged version with only the first four inception modules and a global average pooling, dropout, and one densely connected layer. Mammograms were downscaled to 99 × 99 pixels (general image model) or 299 × 299 (wire localization detection) without preserving aspect ratio. Weights were initialized either randomly (using default Keras settings) or with ImageNet pre-trained weights [8]. For general prediction, we trained all layers, and for prediction of wire localization view, we trained only the last fully connected layer. In order to increase model performance, we applied test-time data augmentation by scoring original and images flipped in horizontal plane. The saliency maps were generated using the LIME module [9].

### Model Selection and Performance Reporting

Hyperparameter tuning for header-based models was performed using fivefold cross-validation within the training set. We report performance of the models on the validation set for further model selection. The performance of our final ensembled model was assessed on the holdout set. We report auROC (area under the receiver operator curve), auPRC (area under the precision-recall curve, also known as average precision score), precision, recall, F1, and accuracy by setting the threshold of 0.5 on prediction score. Significance tests and confidence intervals for auROC were obtained using DeLong’s method as implemented in pROC CRAN package [10]. For comparison of classifier results based on contingency tables, we used exact McNemar’s test as implemented in exact2x2 CRAN package [11].

### Source Code

The source code is available at https://github.com/DSLituiev/mammoviews

## Results

### Header-Based Model

**Fig. 1:**
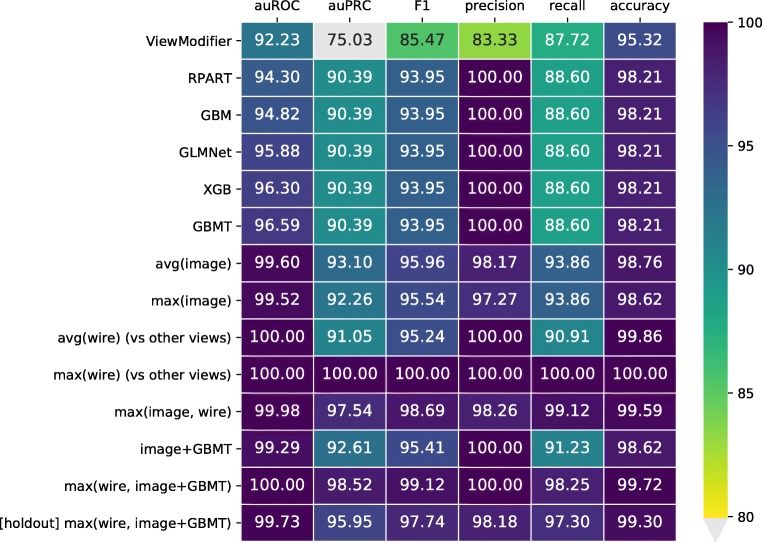
Performance of models and model ensembles. Comparison of machine learning models and their ensembles (rows) is shown according to various metrics (columns). The “wire” row demonstrates performance of the WL model to detect all special views, while “wire (vs other views)” row shows performance of the model to specifically detect WL views. In the last two rows, the performance of the final ensembled model is shown on the validation set and on the holdout set, respectively

**Fig. 2:**
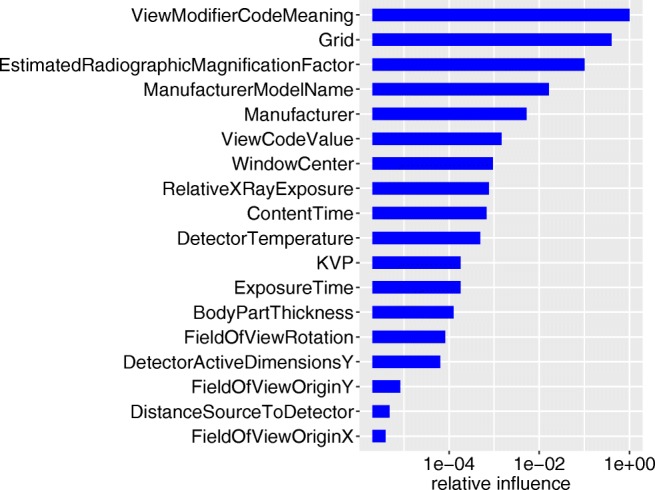
Relative feature influence in GBMT model trained on DICOM headers

We began by training DICOM header-based models. Although all models taken for comparison performed similar within the validation set in terms of accuracy, precision, and recall, the GBMT implementation of gradient boosting machine algorithm performed the best in terms of auROC (96.59%), see Fig. 1a; no significant difference to other header-based models based on the same set of fields). Out of 18 fields picked by the model (see Fig. 2), the “ViewModifierCode” was the most important header field for this model. Thus, we compared the prediction on this field alone to more complex models (Fig. 1) and saw that this field alone performs significantly worse in terms of auROC (92.23%) than all other models except RPART and GBM (Bonferroni-adjusted *p*-value < 0.05), and predictions differ from other header-based models according to McNemar’s chi-squared test (*p*-value = 3.2e-4).

### Image-Based Model

**Fig. 3:**
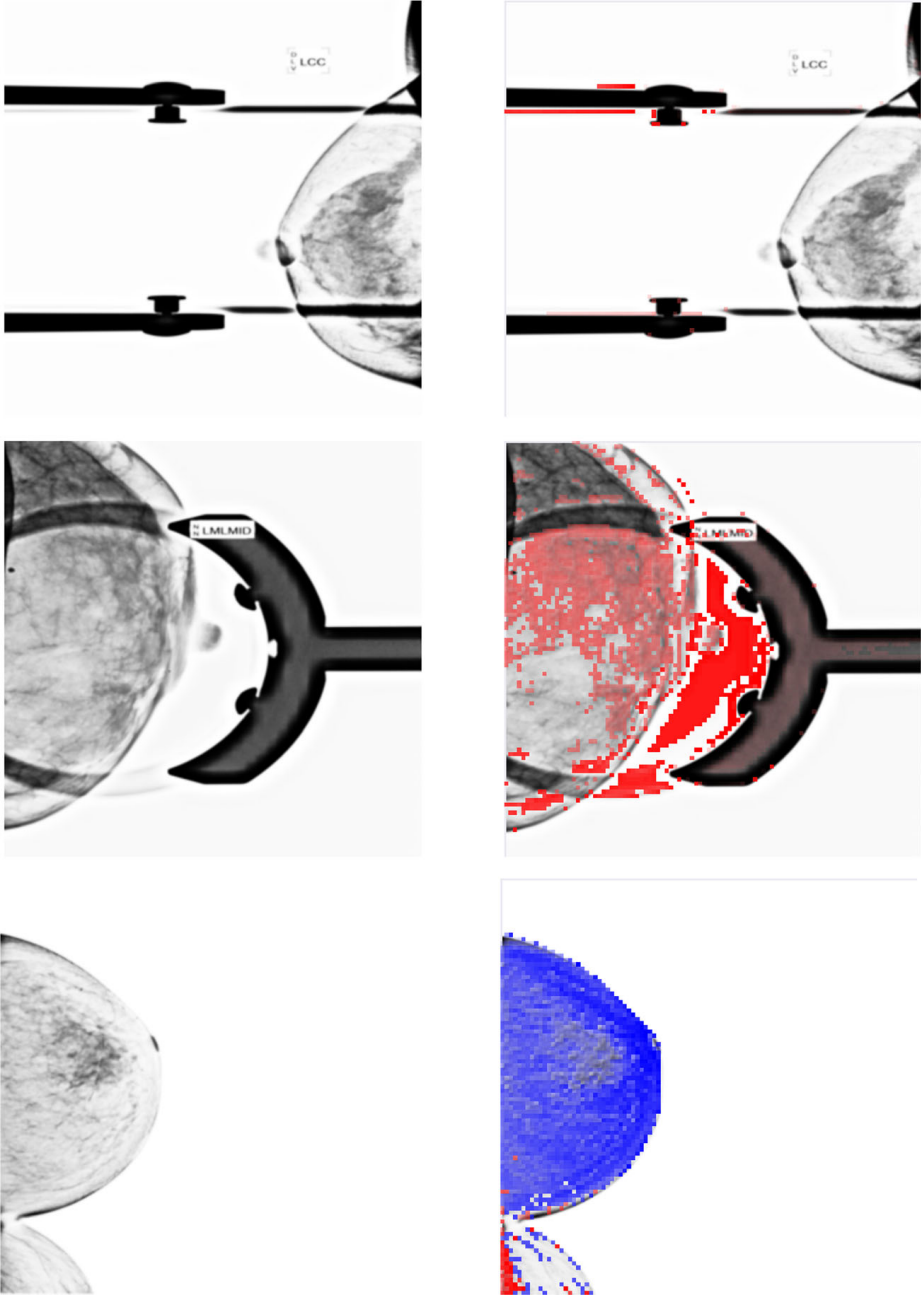
Saliency maps. Two correctly classified spot views and one correctly classified normal view are presented. The region of highest contribution to the “special view” class are highlighted in green and the areas of highest contribution to the “normal view” are highlighted in red

The image model initialized with random weights achieves cross-entropy loss on the validation set of 0.0986 after 25 training epochs and the model initialized with ImageNet pre-trained weights 0.0630 after 55 training epochs. Thus, we further analyzed the model that was initialized with ImageNet weights. In order to increase model performance, we applied test-time data augmentation as described in “Materials and Methods.” Augmented scores were combined by taking their average or maximum, which led to similar auROC results, with averaging performing slightly better. The resulting model achieved an auROC of 99.52%, which is comparable to the highest-scoring header-based model. The saliency maps of three typical model predictions are shown in Fig. 3.

### Image Model for Wire Localization Detection

Analysis of the error patterns for header and image-based models revealed that all of misclassified cases were wire localization (WL) views. This is due to the fact that wire localizations are, technically speaking, a subset of full-field mammograms, and thus, a no distinct view type is recorded in the DICOM headers. Similarly, the CNN model performs poorly on WL images, possibly due to the facts that (1) the wire is a relatively subtle feature and, thus, is harder to detect, and (2) that wire localization DICOMs are underrepresented in the dataset (only 4 WL views out of 4000 total). Therefore, we created a separate image-based classifier for WL views. To this end, we mined 53 images with WL in addition to four images present in the original set (up to 57 total WL images) by matching the keyword “wire” in radiology reports and inspecting obtained matches; these were split proportionally between training, validation, and holdout sets. Next, we trained a separate full inception network with higher resolution input (299 × 299 pixels) to discriminate WL views from regular views. In our final model, only the last fully connected layer was trained, as training all layers resulted in a slight performance degradation. We performed test-time data augmentation by flipping the images in horizontal direction. We compared two ways of combining the augmented scores: by taking mean and maximum of the two scores. We observed that taking maximum of two scores (as compared to taking their mean) improved recall (100.00% vs 92.98%) and the average precision score (100.00% vs 91.05%) in WL detection within the validation set, with the same precision of 100.00% and auROC of 100.00%.

### Combining Models

As a final step, we ensembled the models described above: the header-based model, the general image-based model, and the WL detection model. First, for the purpose of comparison, we combined the general and WL image-based model predictions by taking the maximum of either WL score (obtained by taking maximum score among original and flipped images), or average general image model score (further referred to as “combined image model” or “max(img, wire)” in Fig. 1), which resulted in auROC of 99.98%, thus improving over its predecessors. Next, we combined the image-based model and the GBMT header-based model by taking the mean of the models’ probability scores, denoting the combined prediction as “image + GBMT” (auROC = 99.29%, see Fig. 1 and Fig. S1 and S2 in Online Resource, *p* value = 0.023 and 0.081 in DeLong’s significance test comparison to image-based and GBMT auROC, respectively). Next, we combined this ensembled model with the WL model by taking a maximum score between the “image + GBMT” model and WL model, denoting it as “max(wire, image + GBMT).” The final ensembled model achieved an auROC of 100.00% in the validation set (confusion matrix differs significantly from predictions of GBMT model, but not from the combined image-based model according to McNemar’s test with *p* value = 5.546e-3 and 0.2482, respectively; DeLong’s significance test is undefined for auROC of 100%). Next, the resulting model demonstrated excellent generalization with an auROC of 99.72% on the holdout set, with only 5 out of 715 misclassified cases. Out of five misclassified cases, three were false negatives and two were false positives (see Fig. S3 in Online Resource). Three misclassified special views included one stereotactic view, one WL view, and one specimen view. Out of two misclassified regular views, one had scar markers, thus strongly resembling wire localization view, and the other was a rare auxiliary tail view. Finally, we applied our ensembled model to the unlabeled portion of the dataset (768,178 images). In order to achieve high sensitivity, we used a low threshold of the prediction score of 0.17, which allows to achieve sensitivity of 99.0% on the holdout set and resulted in 10% of images being removed (78,327, including 6114 wire localization views) from the entire image set, leaving 689,851 images of regular mammographic views.

## Discussion

The commoditization of convolutional neural networks and other deep learning architectures and their repeated ability to outperform humans on visual recognition tasks since 2015 [12] makes the availability of training data the most restrictive bottleneck in realizing more precise medical AI. Paradoxically, modern healthcare generates massive volumes of data that can be used to train more precise AI, but it exists largely in unorganized form making it difficult to curate for unbiased large-scale training sets. Here, we demonstrate how we can leverage a range of machine learning techniques and a relatively small labeled set of 4000 images to automatically classify and, thus, pre-filter mammography views in order to facilitate development of a curated imaging dataset from the 772,367 mammography images.

Our analysis of the header-based models showed that most of the fields selected by the model appeared reasonable, as they either explicitly indicate a specific diagnostic protocol such as in “View Modifier Code Sequence,” or physical device parameters such as “Estimated Radiographic Magnification Factor,” “Grid,” “Exposure Time,” and “Relative X-Ray Exposure” which are altered for specialized views. However, we note that some of the weights picked by the header-based model might be specific to this dataset. For example, the predictive value of “Manufacturer” field might be contingent on the processes of the institutions from which our data originates. Mammograms obtained at our institution were taken from only three different manufacturers. However, our dataset also contains referrals from outside institutions which tend to be positive. This leads to a significantly disproportionate representation of outside manufacturers for positive cases. This important caveat suggests that models trained to predict based upon a single institution’s data might perform poorly at another institution whose referral patterns or manufacturer selection differs.

Comparison of the best DICOM header-based model, GBMT to the combined image-based model, showed that the GBMT model performs significantly worse than combined image-based model. Also, the final model incorporating both image- and header-based predictions performs significantly better than the GBMT alone, but only marginally better than combined image-based model. This suggests that the contribution of the header-based model towards performance of the final combined classifier is minor. This is seemingly due to the fact that images carry more reliable and robust information, on which ground truth labels are based.

Although overall performance metrics of our final model were high, several cases were misclassified in the holdout set for some rare views (stereotactic, WL, specimen, and auxiliary tail views). Additionally, we observed a case with scar markers being classified as a special view (due to a high score returned by the WL model), seemingly due to resemblance of the markers to the localization wires. Thus, additional labeled training data might be needed to achieve higher accuracy with these underrepresented image classes. Alternatively, in practical applications, more conservative thresholds can be used to maximize recall by sacrificing precision.

Using the combined model based on both DICOM headers and images allows us to immediately extract and leverage all 772,367 digital mammograms that have been routinely generated at our institution over many years to develop more accurate models of cancer detection that may ultimately help to improve the interpretive performance of breast radiologists. While the generalizability of this approach to other institutions’ data remains unclear, our methods and pipeline can be duplicated elsewhere to allow other large institutions to train their own algorithms towards the direct benefit of patients and ultimate realization of precision mammography for millions of women.

## Electronic supplementary material

ESM 1 (PDF 419 kb)

## References

[1] Berkman Sahiner: Digital Mammography DREAM Challenge Overview (Conference Presentation), Proc. SPIE 10134, Medical Imaging 2017: computer-Aided Diagnosis, 101344I (25 April 2017); doi: 10.1117/12.2280375

[2] Sawyer Lee R, Gimenez F, Hoogi A, Rubin D. Curated Breast Imaging Subset of DDSM. The Cancer Imaging Archive. DOI:10.7937/K9/TCIA.2016.7O02S9CY 2016

[3] Moreira IC, Amaral I, Domingues I, Cardoso A, Cardoso MJ, Cardoso JS (2012). INbreast: toward a full-field digital mammographic database. Acad Radiol.

[4] Madani A, Arnaout R, Mofrad M, Arnaout R: Fast and accurate classification of echocardiograms using deep learning. ArXiv e-prints. 2017; (arXiv:1706) 10.1038/s41746-017-0013-1 PMC6395045 30828647

[5] Mammography Quality Standards Act and Program. 2017 Scorecard statistics. (Accesed on June 5, 2018) https://www.fda.gov/Radiation-EmittingProducts/MammographyQualityStandardsActandProgram/FacilityScorecard/ucm539394.htm

[6] Kuhn M (2008). Building Predictive Models in R Using the caret Package. J Stat Softw.

[7] Friedman J, Hastie T, Tibshirani R: Lasso and elastic-net regularized generalized linear models. R package version, 2011

[8] Szegedy C, Liu W, Jia Y, et al.: Going Deeper With Convolutions. The IEEE Conference on Computer Vision and Pattern Recognition (CVPR), pp. 1–9, 2015

[9] Ribeiro MT, Singh S, Guestrin C.: Why Should I Trust You?: Explaining the Predictions of Any Classifier. arXiv:1602.04938, 2016

[10] Robin X, Turck N, Hainard A (2011). pROC: an open-source package for R and S+ to analyze and compare ROC curves. BMC Bioinformatics.

[11] Dietterich TG (1998). Approximate statistical tests for comparing supervised classification learning algorithms. Neural Computation.

[12] Russakovsky O., Deng J., Su H., Krause J., Satheesh S., Ma S., Huang Z., Karpathy A., Khosla A., Bernstein M., Berg A. C., Fei-Fei L: ImageNet Large Scale Visual Recognition Challenge. arXiv:1409.0575, 2015

