# Supplementary material for: Automatic Labeling of Special Diagnostic Mammography Views from Images and DICOM Headers
Source: J Digit Imaging. 2018 Nov 21;32(2):228–33. doi: 10.1007/s10278-018-0154-z (PMC6456464; doi:10.1007/s10278-018-0154-z)

Electronic Supplementary Material

Automatic labeling of special diagnostic mammography views from images and DICOM headers

Figure 1. DeLong test for difference between pairs of areas under receiver operating curve (auROC).

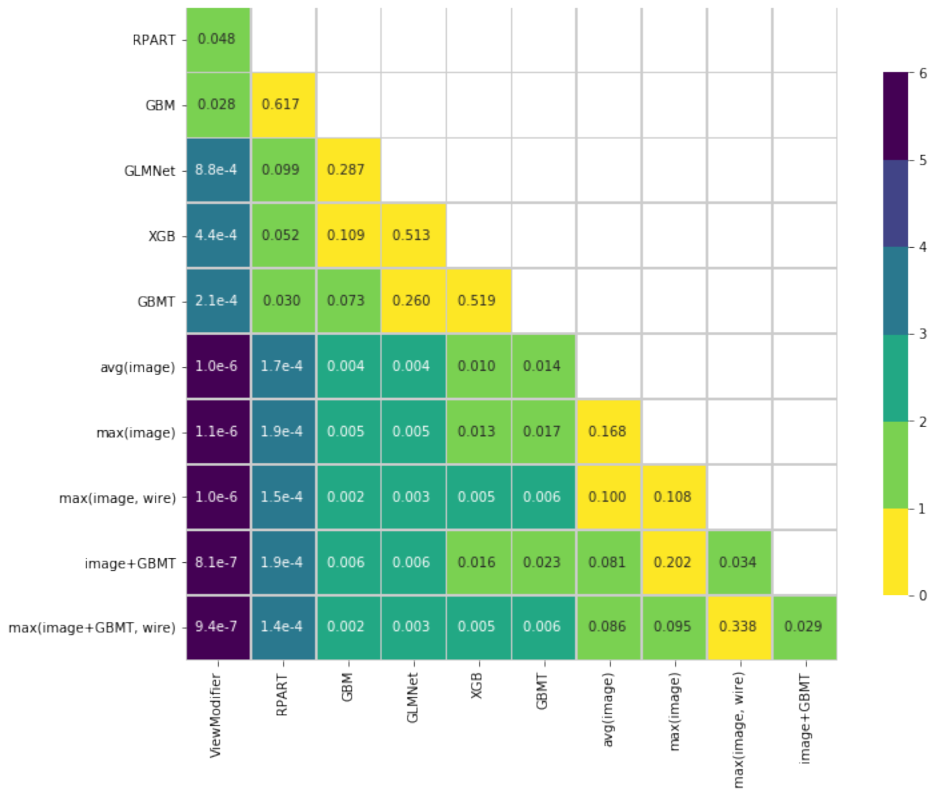

Figure S2. Exact McNemar test for difference in model binary predictions.

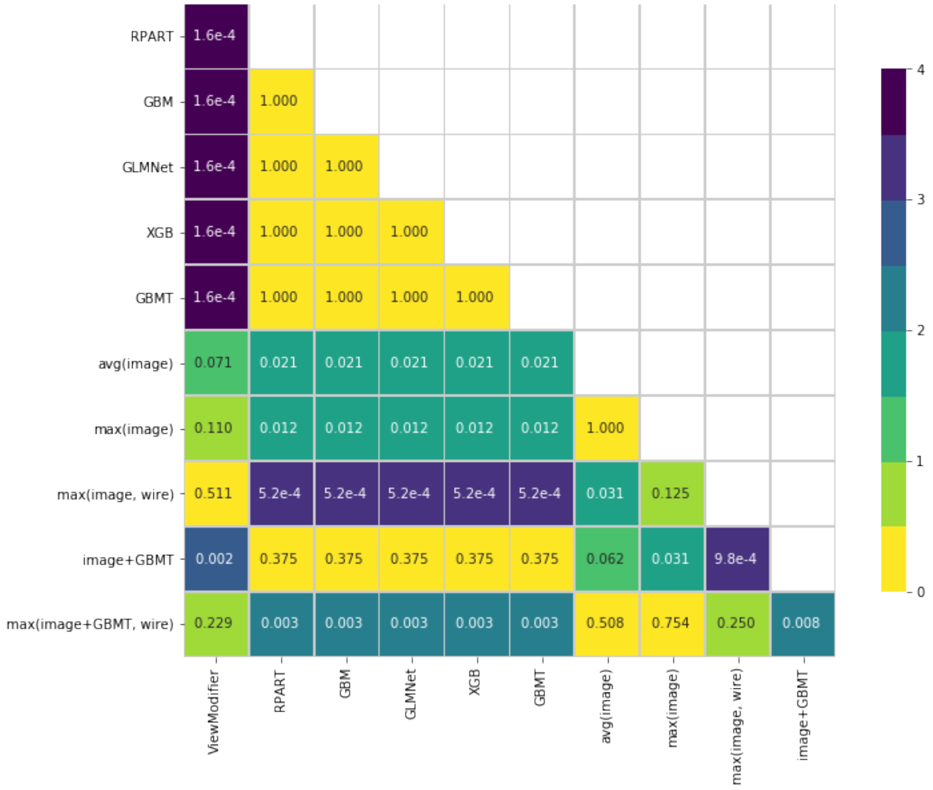

Figure S3. Error analysis. Three false negatives (top panel) and two false positives (bottom panel) present in hold-out set are shown.

A. A stereotactic view with poor contrast classified as normal view

B. A wire localization view classified as normal view.

C. A specimen view classified as normal view

D. A full field view with scar markers classified as special view

E. An axillary tail view classified as special view

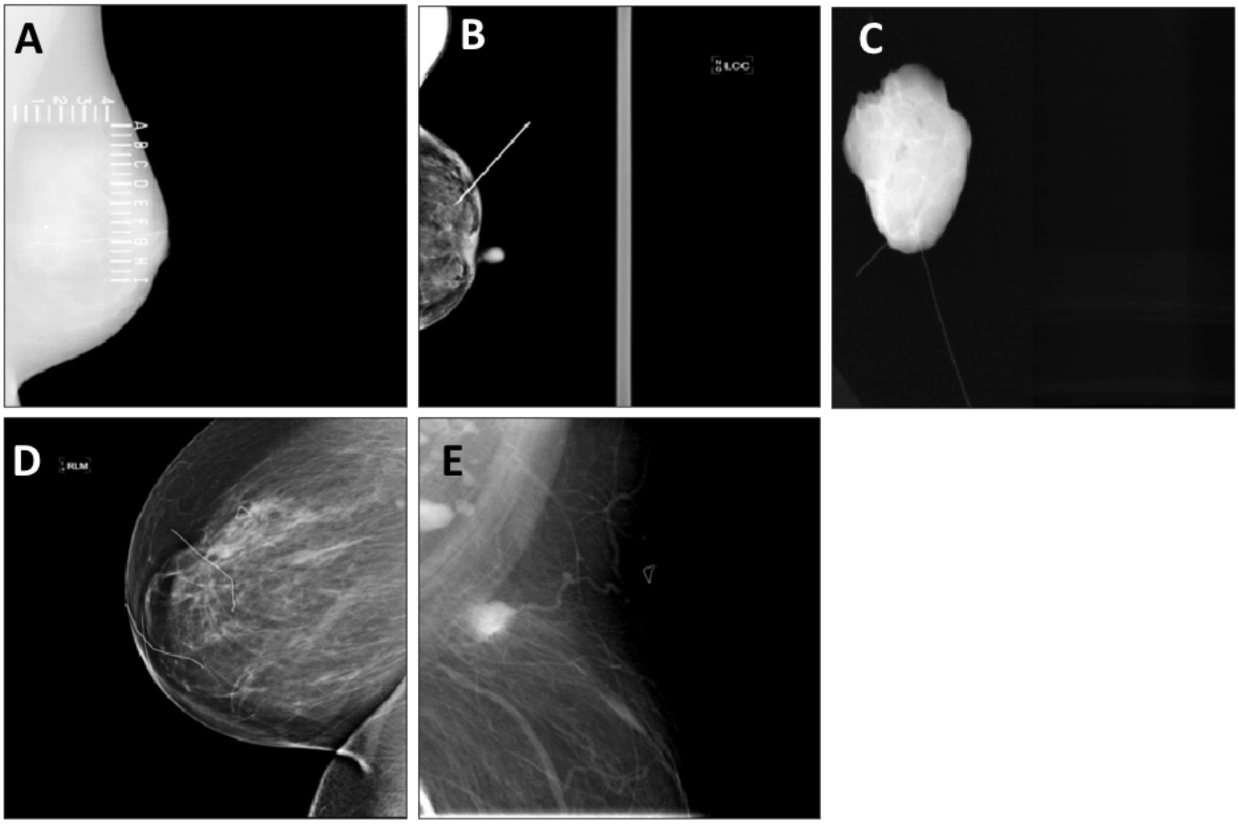

Supplement: Supplementary file 1 — (PDF 419 kb) [file 10278_2018_154_MOESM1_ESM.pdf]
